# Vibration Compensation for a High-Precision Atomic Gravimeter Based on an Improved Whale Optimization Algorithm

**DOI:** 10.3390/s26072133

**Published:** 2026-03-30

**Authors:** Xingyue Guo, Yiyang Zhang, Zhennan Liu, Yi Wang, Shaokai Wang

**Affiliations:** 1 Key Laboratory of Electromagnetic Wave Information Technology and Metrology of Zhejiang Province, College of Information Engineering, China Jiliang University, Hangzhou 310018, China; 2 Division of Time and Frequency Metrology, National Institute of Metrology, Beijing 100029, China; 3 Key Laboratory of State Administration for Market Regulation (Time Frequency and Gravity Primary Standard), Beijing 100029, China; 4 Hefei National Laboratory, Hefei 230088, China; zhennan@hfnl.cn

**Keywords:** cold-atom gravimeter, vibration compensation, whale optimization algorithm, Pearson correlation, gravity measurement sensitivity

## Abstract

**Highlights:**

1. An improved whale optimization algorithm (IWOA) is proposed for vibration compensation, which integrates Logistic-LHS initialization, adaptive search, and Gaussian mutation into the common whale optimization algorithm. 2. With no vibration isolation, IWOA compensation improves the gravity measurement sensitivity by 50% to 47 $\mu \mathrm{Gal}/\sqrt{\mathrm{Hz}}$ when the evolution time *T* is 80 ms. 3. With vibration isolation, IWOA compensation boosts the Pearson correlation between the transition probability and calculated phase to a maximum of 0.98.

**What are the main findings?**

- With no vibration isolation, IWOA minimizes the RMSE of fringe-fitting and outperforms the common coefficient-search method in both speed and accuracy.
- With vibration isolation, IWOA maximizes the Pearson correlation between the atomic signal and calculated phase, outperforming the value of 0.94 reported by Le et al.

**What are the implications of the main findings?**

- The IWOA framework advances fast and accurate algorithmic vibration compensation in cold-atom gravimetry, enabling robust phase restoration and parameter optimization for improved gravity sensitivity.

**Abstract:**

Cold-atom absolute gravimeters are widely used for measuring the acceleration of gravity, yet their sensitivity is often limited by ground vibrations. Existing vibration compensation algorithms struggle to strike a balance between search accuracy and computational efficiency and are prone to local optima. Here, we propose an improved whale optimization algorithm (IWOA) to address these issues. By combining Logistic-LHS (Latin hypercube sampling) chaotic initialization, adaptive adjustment, and a Gaussian mutation operator to prevent premature convergence, IWOA achieves higher search efficiency and superior sensitivity than traditional algorithms. The method is validated through multiple simulation studies and further assessed experimentally on the NIM-AGRb-1 cold-atom gravimeter system. The results show that IWOA reduces the uncertainty of the fitted phase parameter by 66%. The Pearson correlation between atomic transition probability and the calculated phase increases to a maximum of 0.98, and the gravity sensitivity improves to 47 $\mu \mathrm{Gal}/\sqrt{\mathrm{Hz}}$ when the evolution time *T* is 80 ms.

## 1. Introduction

Cold-atom absolute gravimeters (CAGs) are advanced quantum instruments which use cold atoms as the testing mass. They achieve high-precision gravity measurements through matter–wave interference. Since their first application in measuring the gravitational acceleration g in the late 1990s [1], CAGs have progressed rapidly in sensitivity, long-term stability, and systematic error control [2]. They also mitigate wear, drift, and mechanical errors that come from traditional free-fall mechanical gravimeters [3]. In recent years, continuous advances in optical design, laser technology and ultra-high-vacuum engineering have further improved the robustness and operational reliability of CAG systems, enabling absolute gravity measurements in a wider range of geophysical and metrological scenarios while maintaining high accuracy [4,5]. These developments offer important technical support for gravity reference measurements. In quiet laboratory conditions, CAGs can achieve μ precision. However, in noisy environments, ground vibrations may induce fluctuations from μGal to mGal [6], which limits both resolution and sensitivity. As a result, effective suppression of ground vibrations is essential.

Current vibration suppression methods can be divided into two categories: vibration isolation and vibration compensation. Recently, the research on vibration suppression technology has shifted from “single isolator” to a systematic approach of “coordinated optimization of isolation and compensation”. The research on the integration of the two has gradually become an important development direction. Le et al. (2008) [7,8] developed a compact atomic gravimeter, where noise reduction in a low-vibration laboratory environment was achieved using a passive isolation platform and acoustic shielding, combined with IIR and NCLPF filters for interferometric phase compensation. They achieved a correlation coefficient of 0.94 between the transition probability and the calculated phase, and a sensitivity of 14 $\mu \mathrm{Gal}/\sqrt{\mathrm{Hz}}$ at 2*T* = 100 ms. Hauth et al. (2013) [9] suppressed two major systematic effects using a Coriolis compensation scheme and active tilt stabilization of the retroreflection mirror, achieving a sensitivity of 30 $\mu \mathrm{Gal}/\sqrt{\mathrm{Hz}}$ at *T* = 230 ms. Freier et al. (2016) [10] carried out field tests at a high-precision geodetic and astronomical observatory, where ground microseismic noise was suppressed by active isolation combined with seismometer-based post-compensation of the interferometric phase, yielding a sensitivity of 9.6 $\mu \mathrm{Gal}/\sqrt{\mathrm{Hz}}$ at *T* = 260 ms. Huang et al. (2017) [11] tested a home-built ^85^Rb atom absolute gravimeter at both a gravity observatory and a laboratory. By combining passive and active isolation with systematic error correction, they achieved a sensitivity of 30 $\mu \mathrm{Gal}/\sqrt{\mathrm{Hz}}$ at *T* = 200 ms. Wu et al. (2019) [5] used a passive isolation platform and an FPGA-based FIR digital feedback loop to suppress vibrations near the negative-spring resonance, achieving a sensitivity of 37 $\mu \mathrm{Gal}/\sqrt{\mathrm{Hz}}$ at *T* = 130 ms.

Although existing vibration compensation can partially correct residual motion after isolation, it is still limited by model errors, insufficient environmental robustness, and constrained real-time performance. Accordingly, researchers worldwide have pursued further improvements in compensation models and algorithms [12,13,14,15]. Xu et al. (2019) [16,17] employed filtering-based correction in combination with the system frequency response function to calibrate the interferometric signal, enabling compensation of vibration-induced phase shifts exceeding 4π rad. This approach improved gravity measurement sensitivity by more than a factor of two and yielded better results than passive isolation platforms under harsh vibration conditions. Yao et al. (2022) [6] proposed a coefficient-search-based compensation method, which was validated using a cold-atomic gravimeter in an experimental environment without isolation. Under *T* = 80 ms, the standard deviation of the cosine-fit residuals of the interferometric fringes was reduced by 58%. However, its compensation performance did not exhibit a clear advantage over other approaches. Guo et al. (2023) [18] further extended this strategy to different instruments and measurement conditions, achieving a measurement standard deviation of 4.2 μGal and a sensitivity of 18.0 $\mu \mathrm{Gal}/\sqrt{\mathrm{Hz}}$ at *T* = 250 ms. Gong et al. (2023) [19] adopted an improved chaotic sparrow search algorithm, leading to a 70.21% reduction in the modified atom interference fringe fitting uncertainty and a 79.08% decrease in the standard deviation of gravity deviation. Che et al. (2023) [20] introduced the meta-heuristic Equilibrium Optimizer, the mean root-mean-square error reduction reached 37.53% after compensation, and the residual error of gravity measurement reached (34 ± 320) μGal.

However, previous studies mostly focused on the error impact caused by vertical vibration, while neglecting the vibration in the horizontal two-axis directions and the experimental environment was relatively monotonous. In an experimental environment similar to that of Yao et al., this work proposes a vibration compensation scheme for a cold-atomic gravimeter, addressing the limitations of their method in terms of high computational cost and long computation time. The proposed scheme is centered on an improved whale optimization algorithm (IWOA). By incorporating Logistic-LHS chaotic initialization, Gaussian perturbation, and an adaptive adjustment strategy, the IWOA searches for the optimal combination of gain coefficients and delay parameters, thereby achieving better vibration compensation. We take into account the vibrations along the two horizontal axes and conduct experiments using the laboratory-developed NIM-AGRb-1 [21] cold-atom gravimeter under both passively isolated and non-isolated conditions. The effectiveness of the proposed algorithm is first validated through analyses of multiple simulated datasets. Comparative experiments are performed to evaluate the performance of IWOA against several alternative algorithms under identical conditions. Performance differences are assessed using metrics such as RMSE, fitted phase parameter uncertainty, Allan deviation and Pearson coefficient.

The remainder of this paper is organized as follows: Section 2 introduces the principles of vibration compensation for atomic gravimeters and outlines the IWOA methodology. Section 3 presents simulation studies to validate the effectiveness of IWOA. Section 4 analyzes the experimental results and compares the compensation performance of different algorithms. Section 5 concludes the paper and discusses prospects for optimizing vibration compensation.

## 2. Principles and Methods

### 2.1. Vibration Compensation Method

**Figure 1:**
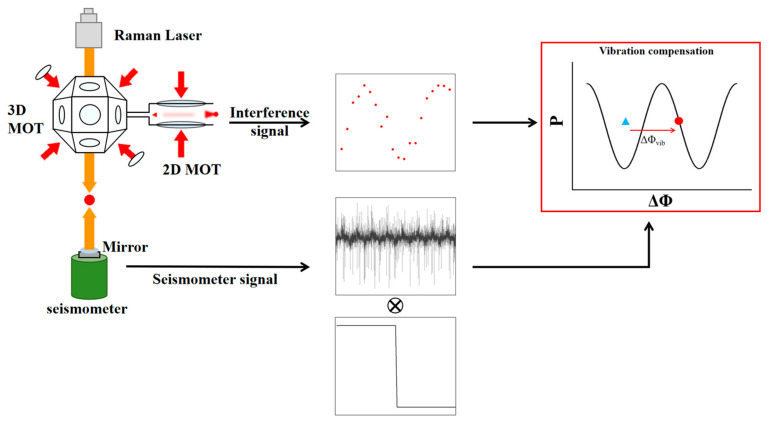
Vibration Compensation Process. It shows the simultaneous recording of atomic signals (red dots) and seismometer signals, the process of convolving vibration signals with the gravimeter sensitivity function to derive the phase, and vibration compensation.

The principle of vibration compensation is illustrated in Figure 1. The seismometer and atomic signals are recorded simultaneously, and after preprocessing steps such as filtering and reconstruction, the seismometer signal is convolved with the gravimeter sensitivity function to derive the vibration-induced phase shift. This term is then subtracted from the measured phase, thereby accomplishing the compensation.

The measured atomic interference fringes *P* satisfy Equations (1) and (2)

(1) $$P=\frac{1}{2}\left(1+B\cos\Delta \Phi \right)$$

(2) $$\Delta \Phi =\left(k_{eff}\cdot g-\alpha \right)T^{2}+\Delta \varphi_{vib}+\Delta \varphi_{others}$$

where *B* represents the contrast of an atomic interference fringe, ΔΦ is the atomic interference phase; $\Delta \varphi_{vib}$ is the interference phase caused by vibration noise, which is the term that requires vibration compensation; $\;\Delta \varphi_{others}$ is the interference phase caused by other noise sources; *α* is the sweep frequency rate; $k_{eff}$ is the effective wave vector of Raman light; and *T* is the evolution time between two adjacent Raman light pulses. When the atomic gravimeter operates at the center of the cosine ramp, the interference signal and the change in phase exhibit a nearly linear relationship, as shown in Equation (3), indicating that a small phase shift can cause a significant change in the signal

(3) $$\frac{dP}{d\Delta \Phi }=\frac{B\cdot \sin\Delta \Phi }{2}$$

when Φ=±π/2 at the center of the slope, ${\left|dP/d\Delta \Phi \right|}_{\max}=B/2$. The sensitivity is at its maximum and the relationship is approximately linear, enabling linear mapping between phase and gravity changes.

**Figure 2:**
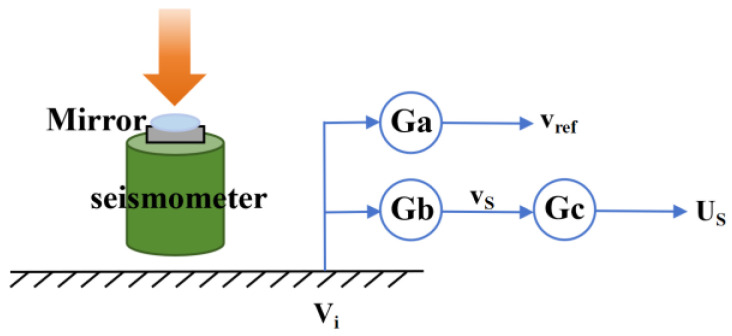
The relationship between the output signal of the seismometer and the real vibration of the reference mirror. It includes the transmission relationship among the mirror vibration, ground vibration, the vibration of the seismometer’s sensitive mass, and the seismometer output.

In the experiment, the Raman reflector is rigidly connected to the seismometer, and the seismometer is placed on the ground. Therefore, there exists a transfer function between the ground, the seismometer, and the Raman reflector, meaning the signal output by the seismometer does not fully reflect the true vibration of the reflector. This relationship can be explained by Figure 2. Here, Ga represents the transfer function between the ground vibration velocity *V_i_* and the reflector vibration velocity *v_ref_*, Gb denotes the transfer function between the ground vibration velocity *V_i_* and the seismometer’s sensitive mass vibration velocity *v_s_*, and Gc refers to the transfer function between the sensitive mass motion velocity *v_s_* and the seismometer output signal *U_s_*.

However, in actual measurements, due to complex factors such as seismic propagation paths and the internal mechanical structure of the seismometer, Ga and Gb are difficult to measure directly, and Gc is not an ideal linear transfer function and may vary with measurement time and environmental changes. Since the measurement environment in a single gravity measurement experiment does not change significantly and the measurement time generally does not exceed 24 h, a simplified transfer function model consisting of gain coefficient *K* and delay coefficient *τ* is introduced as Equation (4) [6,16]:

(4) $$v_{ref}\left(t\right)=K\cdot v_{s}\left(t+\tau \right)=\frac{1}{K_{S}}\cdot K\cdot U_{S}\left(t+\tau \right)\;$$

where *t* is time and K_S_ is the nominal sensitivity of the seismometer, which can be found in the instrument operation manual. The phase shift caused by the vibration of the Raman mirror is calculated as Equation (5):

(5) $$\Delta \varphi_{vib}=k_{eff}\cdot \int_{T}^{-T}g_{s}\left(t\right)v_{ref}\left(t\right)dt=k_{eff}\cdot \frac{1}{K_{S}}\cdot K\cdot \int_{T}^{-T}g_{s}\left(t\right)U_{S}\left(t+\tau \right)dt\;$$

where *g_s_(t)* is the sensitivity function of the atomic gravimeter with π/2−π−π/2 Raman pulses, which is expressed as Equation (6):

(6) $$g_{s}\left(t\right)=\left\{\begin{array}{cc} -1 & -T\leq t<0\\ 1 & 0\leq t\leq T \end{array}\right.$$

Theoretically, the closer the gain coefficient *K* and the delay coefficient *τ* are to the actual values, the more accurately they can reflect the actual vibration of the mirror.

### 2.2. Improved Whale Optimization Algorithm (IWOA)

In the context of vibration compensation for atomic gravimeters, the algorithm must balance global optimization capability with local search precision to accurately reconstruction the actual vibration phase. Existing commonly used optimization algorithms each have their respective strengths and limitations. Although the black widow optimization algorithm shows potential, it lacks sufficient validation in this field and its stability remains uncertain [22]. The gradient descent algorithm offers high local search precision but is highly susceptible to falling into local optima [23]. Stochastic algorithms provide broad global coverage but suffer from slow convergence and unstable solutions [24]. Therefore, to simultaneously meet the dual requirements of global optimization capability and local search precision in vibration compensation problems, the whale optimization algorithm (WOA) [25], by simulating the encircling prey, bubble-net attacking, and random search mechanisms of humpback whales, naturally achieves a dynamic balance between global exploration and local exploitation.

Encircling prey is an active search behavior of whales. After knowing the location of the prey, the whale group updates their positions based on the relationship between their own positions and the location of the target prey. The behavior is expressed by the following Equations (7) and (8) as

(7) $$\vec{X}\left(\mathrm{iter}+1\right)=\vec{X^{*}}\left(iter\right)-\vec{A}\cdot \vec{D}$$

(8) $$\vec{D}=\left|\vec{C}\cdot \vec{X^{*}}\left(\mathrm{iter}\right)-\vec{X}\left(\mathrm{iter}\right)\right|$$

where *iter* is the current iteration, and $\vec{A}$ and $\vec{C}$ are coefficient vectors, $\vec{X^{*}}\left(\mathrm{iter}\right)$ is the position vector of the current optimal solution, $\vec{X}\left(\mathrm{iter}\right)$ is the current position vector, and $\vec{X}\left(\mathrm{iter}+1\right)$ is the new generation position vector. The sigmoid function and adaptive function are introduced to regulate the vectors, in order to dynamically balance the global search ability and local exploration ability of the whale optimization algorithm. The calculation formulas for vectors $\vec{A}$ and $\vec{C}$ are expressed by Equations (9)–(12) as

(9) $$\vec{A}=2\left({\vec{a}}_{Sigmoid}+{\vec{a}}_{Adaptive}\right)\cdot \vec{r}-\left({\vec{a}}_{Sigmoid}+{\vec{a}}_{Adaptive}\right)\;$$

(10) $${\vec{a}}_{Sigmoid}=\frac{1}{1+\exp\left[-k\left(iter-\frac{iter_{\mathrm{Max}}}{2}\right)\right]}$$

(11) $${\vec{a}}_{Adaptive}=\vec{a}\cdot (1-\frac{iter}{iter_{\mathrm{Max}}})\;$$

(12) $$\vec{C}=2\cdot \vec{r}$$

where $\vec{a}$ decreases linearly from 2 to 0 during the iteration process. $\vec{r}$ is a random vector in [0, 1], and k is the steepness of the sigmoid function. ${\vec{a}}_{Sigmoid}$ and ${\vec{a}}_{Adaptive}$ are the sigmoid function and the adaptive function, respectively, which are used to regulate the vector $\vec{A}$. The process of the bubble-net attacking method involves establishing a spiral equation between the positions of the whale and the prey, the behavior is expressed by the following Equations (13) and (14) as

(13) $$\vec{X}\left(iter+1\right)=\vec{D^{\prime }}\cdot \exp\left(cl\right)\cdot \cos\left(2\pi l\right)+\vec{X^{*}}\left(\mathrm{iter}\right)\;$$

(14) $$\vec{D\prime }=\left|\vec{X^{*}}\left(iter\right)-\vec{X}\left(iter\right)\right|$$

where $\vec{D\prime }$ is the distance from the i-th whale to the prey, c is a constant used to define the spiral shape, and l is a random number in [−1, 1]. By introducing a probability parameter p to randomly select either the behavior of encircling prey or the bubble-net attacking method, the mathematical model is expressed by the following Equation (15):

(15) $$\vec{X}\left(iter+1\right)=\left\{\begin{array}{cc} \vec{X^{*}}\left(iter\right)-\vec{A}\cdot \vec{D} & ,\mathrm{if}\;p<0.5\\ \vec{D\prime }\cdot e^{cl}\cdot \cos\left(2\pi l\right)+\vec{X^{*}}\left(iter\right) & ,\mathrm{if}\;p\geq 0.5 \end{array}\right.\;$$

The adaptive change of search vector A enables the algorithm to smoothly transition between development and search. When |$\vec{A}$| > 1, the algorithm focuses on search, while when it is less than 1, it is used for development. The mathematical model of this behavior is expressed by the following Equations (16) and (17) as

(16) $$\vec{X}\left(iter+1\right)={\vec{X}}_{rand}\left(iter\right)-\vec{A}\cdot \vec{D}$$

(17) $$\vec{D}=\left|\vec{C}\cdot {\vec{X}}_{rand}\left(iter\right)-\vec{X}\left(iter\right)\right|$$

where ${\vec{X}}_{rand}\left(iter\right)$ is the random position vector selected from the current population.

The original WOA is highly sensitive to the initial population distribution. Random initialization often leads to uneven individual distribution and insufficient coverage, which limits the global search performance. Therefore, a Logistic-LHS hybrid initialization strategy combines the global traversal of logistic mapping with the spatial uniformity of Latin hypercube sampling, generating evenly distributed individuals to improve population quality and enhance global search capability and stability.

Split the population of size $N_{particles}$ into two subsets: $N_{logistic}=\beta \cdot N_{particles}$ individuals are initialized using the Logistic map, and $N_{lhs}=\left(1-\beta \right)\cdot N_{particles}$ individuals are initialized using Latin hypercube sampling. The two subsets are initialized independently and then concatenated to form the initial population. The Logistic-LHS chaotic mapping formula is shown in Equation (18)

(18) $$y_{i}=\left\{\begin{array}{cc} y_{i}^{logistic}=a+\left(b-a\right)\cdot x_{i} & ,if\;i\leq N_{logistic}\\ y_{i}^{lhs}=a+m\cdot \Delta x+rand\cdot \Delta x & ,if\;i>N_{logistic} \end{array}\right.\;$$

where a and b represent the upper and lower limits of the parameters, *x_i_* is $R\cdot x_{i-1}\cdot \left(1-x_{i-1}\right)$, R is the chaotic parameter of the Logistic mapping. Δ*x* is $\frac{b-a}{N_{lhs}}$, and *rand* is a random number in [0, 1),$\;m=0,1,\ldots \;,N_{lhs}-1$.

Furthermore, we propose the Gaussian distribution mutation operator, which randomly mutates the updated positions of the whales to prevent the optimization process from getting stuck in local optima. The updated position with perturbation is expressed by the following Equation (19) as:

(19) $$\vec{X}\left(iter+1\right)=\vec{X}\left(iter\right)+N\left(0,{\vec{a}}_{Sigmoid}\right)\;$$

**Figure 3:**
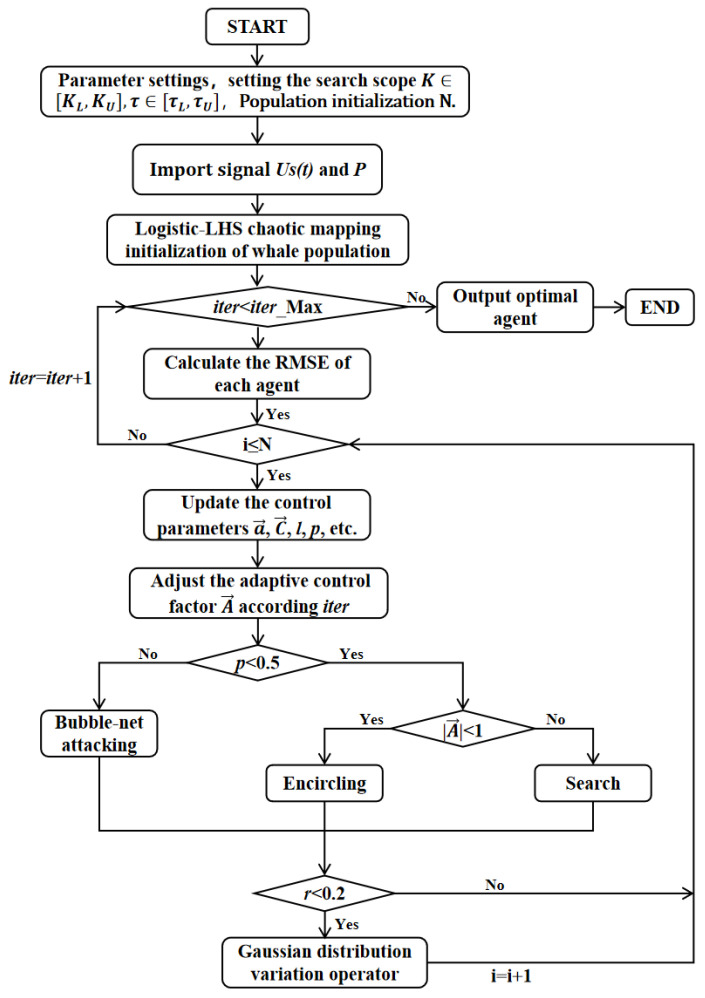
Flowchart of the IWOA algorithm. It includes data import, data preprocessing, IWOA search for the optimal coefficients, and result output sections.

The flowchart of the IWOA algorithm is illustrated in Figure 3:

The pseudo-code of the IWOA Algorithm 1 is as follows:
**Algorithm 1. Pseudo-code of Improved Whale Optimization Algorithm (IWOA)**1. $\mathrm{Set}\;\mathrm{the}\;\mathrm{parameters}\;\mathrm{and}\;\mathrm{import}\;\mathrm{signal}\;U_{S}\left(t\right)$ and *P*2. Initialize the whales population with Logistic-LHS strategy by **Equation (18)**3. **While *iter* < *iter*_Max**4.      **For**
5.      Calculate the RMSE of all agents and select the optimal agent6.      **End for**7.      **For *i* ≤ N**8.      $\mathrm{Update}\;\mathrm{the}\;\mathrm{control}\;\mathrm{parameters}\;\;\vec{a}$$,\;\;\vec{C}$, *l* and *p*9.      $\mathrm{Adjust}\;\mathrm{the}\;\mathrm{adaptive}\;\mathrm{control}\;\mathrm{factor}\;\vec{A}$ according *iter* by **Equations (9)–(11)**10.            **If1 *p* < 0.5**11.                  $\mathrm{If}2\;|\vec{A}|$** < 1**12.                  Update the position of the current search agent by **Equation (7)**13.                  $\mathrm{Else}\;\mathrm{if}2\;|\vec{A}|$** ≥ 1**14.                  $\mathrm{Select}\;a\;\mathrm{random}\;\mathrm{search}\;\mathrm{agent}\;{\vec{X}}_{\mathrm{rand}}$15.                  Update the position of the current search agent by **Equation (16)**16.                  **End if2**17.            **Else if1 *p* ≥ 0.5**18.            Update the position of the current search by **Equation (13)**19.            **End if1**20.            **If3 *r* < 0.2**21.            Add Gaussian distribution variation operator by **Equation (19)**22.            **End if3**23.      **End for**24.      Check if any search agent goes beyond the search space and amend it25.      $\mathrm{Update}\;\vec{X}\left(\mathrm{iter}+1\right)$ if there is a better solution26.      *iter = iter* + 127. **End while**28. $\mathrm{return}\;\vec{X}\left(\mathrm{iter}+1\right)$

## 3. Simulation

**Figure 4:**
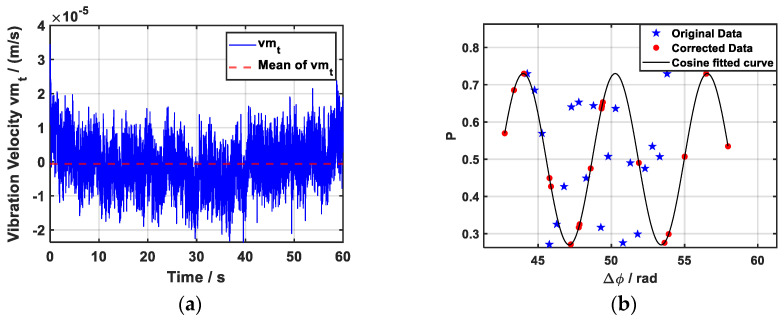
(**a**) Simulated vibration signal generated based on the global seismic background noise, has an average value of zero within 60 s. (**b**) The interference fringes before and after the simulation signal correction and the fitting curve. The simulated vibration noise is nearly eliminated after correction, and the fringes are much closer to the theoretical fitted curve.

Before experimental measurements, simulations are essential for verifying the effectiveness of the algorithm. The simulation procedure is as follows: (1) Based on the control parameters of the gravimeter, set appropriate values for the relevant parameters, including *k_eff_*, *g_s_(t)*, *T* and *α*. (2) Using the global seismic background noise models, namely the New Low-Noise Model (NLNM) and New High-Noise Model (NHNM) [26], generate the simulated vibration voltage output of the seismometer, as illustrated in Figure 4a. (3) Set the gain coefficient K_set_ = −1 and delay coefficient τ_set_ = 5 ms, calculate the actual phase shift introduced by the vibration of the Raman mirror according to Equations (4)–(6), and then, using Equations (1) and (2), compute the atomic transition probability *P_sim_* in the absence of additional phase noise, which serves as the simulated output of the atomic gravimeter. (4) The vibration compensation algorithm is then validated using the simulated *P_sim_* together with the corresponding vibration data *U_S_*(*t*).

**Figure 5:**
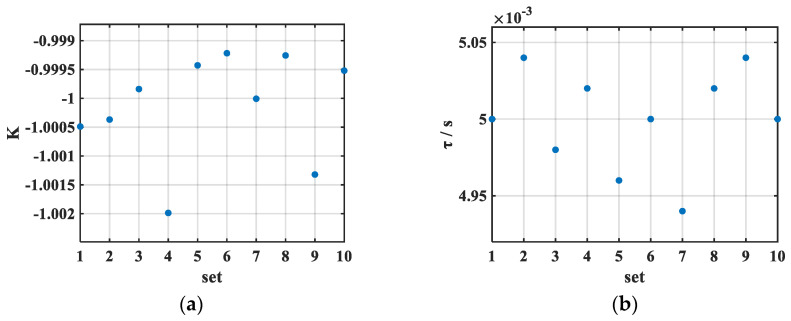
Optimal parameters obtained by the IWOA vibration compensation algorithm: (**a**) gain factor and (**b**) delay factor. IWOA performed optimal parameter search on simulated atomic and seismometer signals; the resulting optimal *K* and *τ* values closely matched the preset ones, demonstrating the effectiveness of the algorithm.

Ten sets of simulated interferometric fringe sequences and vibration velocities were used as inputs for the Algorithm 1 for analysis. The resulting gain and delay coefficients of the compensated fringe sequences are shown in Figure 5a,b. The mean value of *K* was −1 with a standard deviation of 0.87 × 10^−3^, while the mean delay *τ* was 5 ms with a standard deviation of 0.03 × 10^−3^ s. Before compensation, the cosine fit to the atomic interference fringes yielded an RMSE of 165.17 × 10^−3^, whereas after compensation, the mean RMSE was reduced to only 0.27 × 10^−3^. These results demonstrate that the algorithm is capable of accurately recovering the pre-defined parameters and achieving effective vibration compensation. As illustrated in Figure 4b, the compensated fringes exhibit better agreement with the fitted cosine curve than the original fringes, thereby confirming the validity of the proposed compensation method.

## 4. Experiments

### 4.1. Experiment Setup

**Figure 6:**
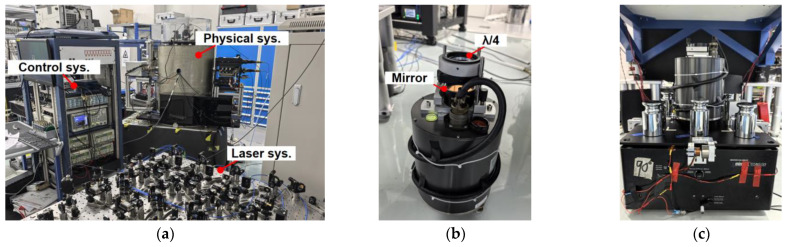
(**a**) Experimental setup consisting of the control system, laser system, and physics package; (**b**) Güralp Systems Ltd. 3ESPC seismometer (Reading, United Kingdom) employed in the laboratory; (**c**) Minus-K 50BM-4 vibration isolation platform (Inglewood, CA, United States).

The proposed vibration compensation method based on IWOA was experimentally implemented on the home-built NIM-AGRb-1 cold-atomic gravimeter [19], with the experimental setup shown in Figure 6a,b. A broadband seismometer (3ESPC, Güralp Systems Ltd. Reading, United Kingdom) was positioned directly beneath the Raman retro-reflection mirror to monitor its vibrations. The instrument provides an effective bandwidth of 0.033–50 Hz and a sensitivity of 2000 V/(m/s). The Raman mirror was aligned directly below the interferometer region, with its vibration direction coinciding with the seismometer’s measurement axis to ensure sensitivity.

**Figure 7:**
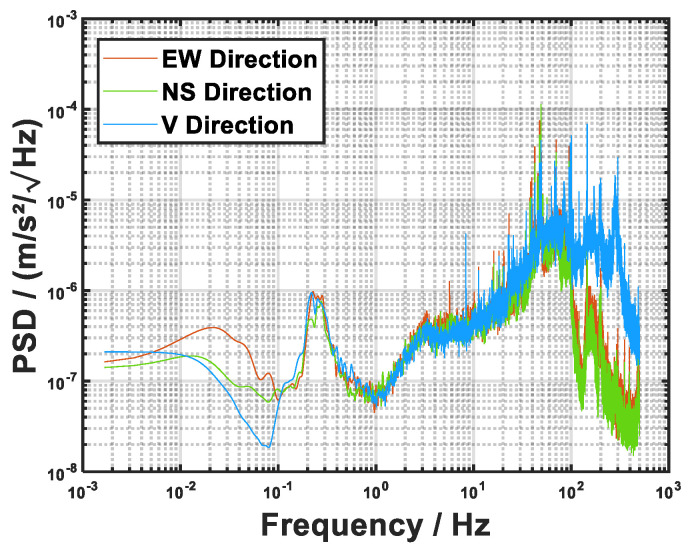
The power spectral density of the vertical, east-southern, and north-western ground vibration acceleration at the test site.

During the free fall of the cold-atom ensemble in the detection chamber, the time interval between laser pulses was set to *T* = 80 ms, with a laser wavelength of approximately 780 nm. A linear chirp of the laser frequency was applied to obtain the interference fringes. The vibration signal recorded by the seismometer was advanced by 75 ms relative to the first laser pulse in the atomic free fall, ensuring synchronization between the sampled seismic data and the interferometric sequence. The experiments were conducted in a underground laboratory (second basement level) with good seismic isolation conditions, and the power spectral density of the ground vibration acceleration at the test site is shown in Figure 7.

In this experiment, data from 30 measurement cycles were collected, with each gravity measurement cycle consisting of 20 free falls of atomic ensembles. During each cycle, the vibration velocity signal from the seismometer, corresponding to the timing of the gravity measurement, was recorded at a sampling rate of 50 kHz. The seismometer output was voltage, denoted as Ui(t), i = 1,2,…,30. The acquired atomic signals were synchronized with the seismometer signals, and a vibration compensation algorithm was applied for post-processing correction. In the proposed approach, the IWOA position parameters were defined as the gain and delay coefficients to be optimized, thereby reducing the transfer function problem to a two-variable optimization task.

### 4.2. Results

**Figure 8:**
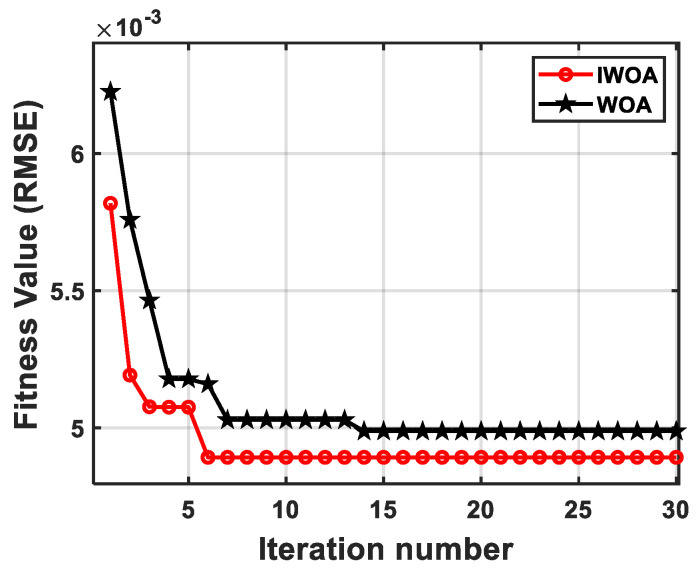
The iterative curves of the IWOA and WOA optimization transfer function models. IWOA reached the optimal fitness value after the 6th iteration and WOA after the 13th.

The performance of the IWOA algorithm was analyzed and validated on the MATLAB R2022b platform. The whale population size was set to 50, with a maximum of 30 iterations. The search ranges for the gain and delay coefficients were [−1.5, 1.5] and [0, 0.12], respectively. As shown by the iterative optimization curve in Figure 8, the initial RMSE of WOA was relatively large and reached an optimal solution after the 13th iteration, yielding a minimum RMSE of 5.00 × 10^−3^. The IWOA reached an optimal solution after the 6th iteration, yielding a minimum RMSE of 4.89 × 10^−3^ with the optimal gain coefficient $K_{\mathrm{opt}}$ = −1.2 and the optimal delay coefficient $\tau_{\mathrm{opt}}$ = 6.3 ms. In contrast, the RMSE without vibration compensation was 11.43 × 10^−3^. Compared with the previous WOA, IWOA has a faster convergence speed and higher accuracy. These results demonstrate that IWOA exhibits rapid convergence during transfer function parameter optimization, efficiently identifying the optimal parameter set. After performing vibration compensation on multiple sets of measured data, when the compensation effect reaches its optimum, the interval of the optimal gain coefficient $K_{opt}$ is [−0.977, −1.244] and the interval of the optimal delay coefficient $\tau_{opt}$ (s) is [0.00002, 0.01626].

**Figure 9:**
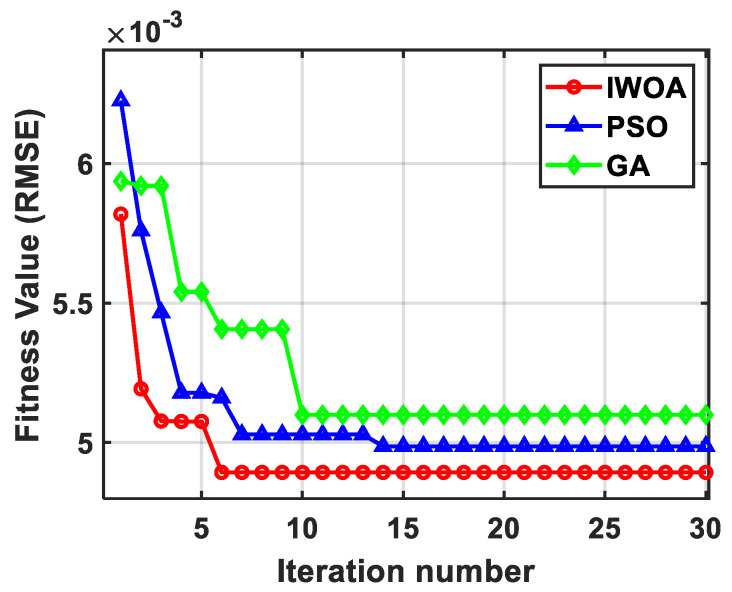
Evaluation of parameter optimization results of the simplified transfer function model across various algorithms. IWOA reached the optimal fitness value after the 6th iteration (best), PSO after the 14th, and GA after the 10th (worst).

To assess the performance advantage of IWOA, comparative experiments were conducted with two alternative optimization algorithms: particle swarm optimization (PSO) and genetic algorithm (GA). For each single vibration-compensation run, the convergence rate was evaluated through the iterative optimization curves, while search performance was assessed using the minimum fitness value and the RMSE reduction ratio. All three algorithms were executed with the same population size and maximum iteration number, and the comparison results are presented in Figure 9. The IWOA, PSO, and GA algorithms converged after the 6th, 14th, and 10th iterations, respectively, with minimum fitness values of 4.89 × 10^−3^, 4.99 × 10^−3^, and 5.10 × 10^−3^. As illustrated in the figure, compared with GA, IWOA converges much faster during the early iterations, enabling a faster convergence toward the optimal solution. Relative to PSO, IWOA exhibits smaller post-convergence fluctuations, reflecting stronger global optimization capability and avoiding oscillatory behavior. Overall, IWOA surpasses both PSO and GA in convergence speed and optimization sensitivity, highlighting its superior performance.

**Figure 10:**
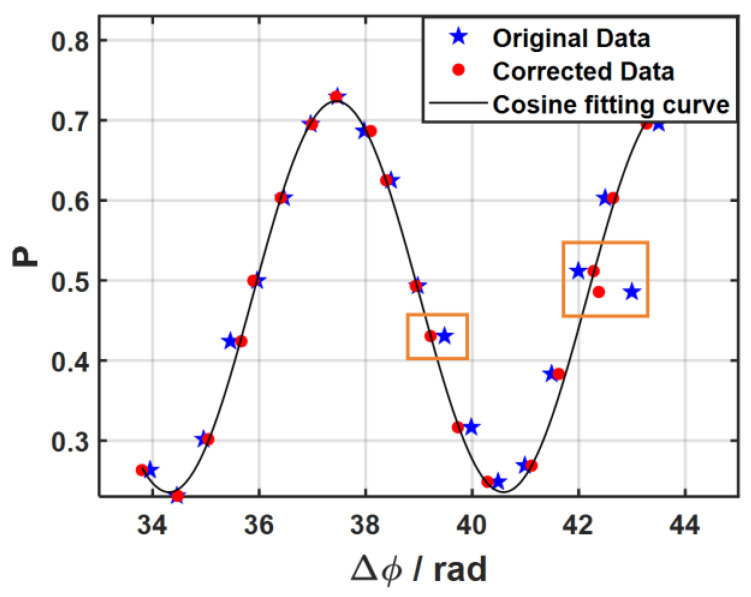
The interference fringes before and after correction and the theoretical cosine fitting curve (typical correction points are marked with orange boxes).

A comparison of the atomic interference fringes before and after vibration compensation is shown in Figure 10. The compensated fringes align more closely with the fitted cosine curve, although residual deviations remain. These deviations arise primarily from two factors: (i) Seismometer performance limitations lead to amplitude and phase distortions in its response function, while horizontal vibrations coupling into the vertical signal introduce additional noise. (ii) In addition to the vibration interference of the gravimeter, the laser phase noise and various noise sources in the atomic detection introduce further error.

Three vibration compensation approaches—coefficient search, genetic algorithm (GA) and improved whale optimization algorithm (IWOA)—were compared with respect to computation time, fitted phase parameter uncertainty, and gravity measurement uncertainty. The single-measurement comparison results are summarized in Table 1. The fitted phase parameter uncertainty of the compensated interferometric fringes were reduced by 45%, 54%, and 66%, respectively. With IWOA compensation, the gravity measurement uncertainty was reduced threefold, while the computation time was only 15 s. Compared with the coefficient search and GA approaches, IWOA yielded a substantial enhancement in both fitting quality and gravity measurement sensitivity, thereby demonstrating its effectiveness for vibration compensation in an atomic interferometric gravimeter.

**Figure 11:**
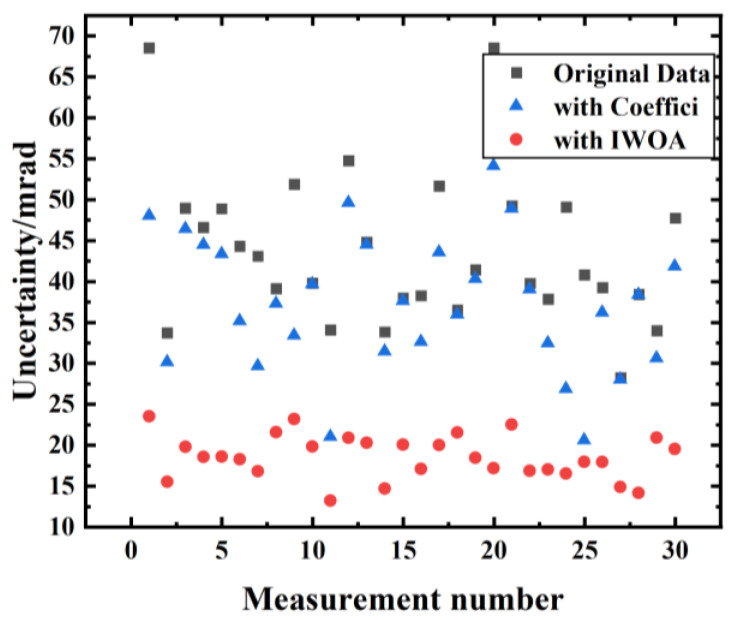
Distribution of fitted phase uncertainties across 30 measurements. The coefficient search algorithm results in highly fluctuating fitted phase parameter uncertainty after vibration compensation, peaking above 50 mrad, whereas IWOA achieves the smallest variation, with all uncertainties under 25 mrad.

Figure 11 shows the distribution of phase fitting uncertainties over 30 measurements obtained by the coefficient search algorithm and the IWOA method. The original fringe fits exhibited pronounced fluctuations, with large uncertainties reaching a maximum of 68 mrad. While the coefficient search algorithm partially suppressed these uncertainties, significant variations remained. In contrast, the IWOA method markedly reduced the fitted phase uncertainties to below 25 mrad, with minimal variation, highlighting its superior stability and robustness.

**Figure 12:**
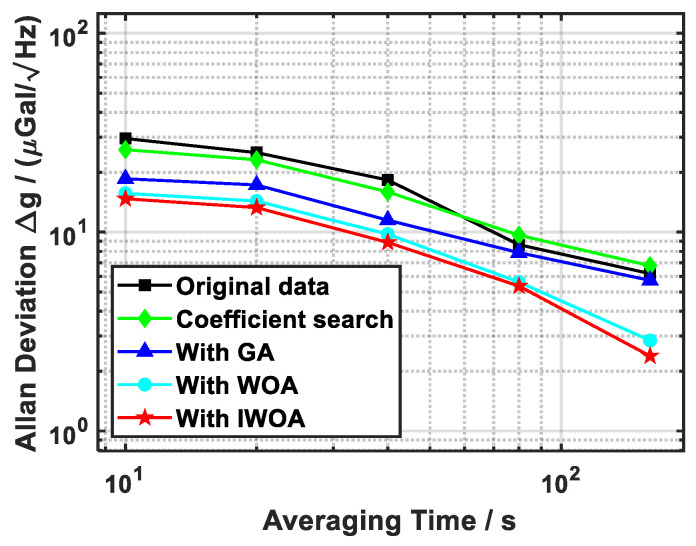
Evaluation of Δg Allan deviation across multiple methods. The IWOA method achieved superior gravity measurement sensitivity compared to other methods while maintaining a smaller error bar.

To further assess the effect of vibration compensation algorithms on gravity measurements, the Allan standard deviation of the measured gravity acceleration was calculated. Given the short duration of the measurement, solid Earth tide corrections were not applied. As shown in Figure 12 and listed in Table 2, the gravity measurement resolutions obtained with compensation using the coefficient search algorithm, GA, WOA and IWOA were 7 μGal@160s, 6 μGal@160s, 3 μGal@160s and 2 μGal@160s, respectively. Relative to the original uncompensated data, IWOA achieved a 62% improvement in resolution, clearly outperforming both the coefficient search and GA methods. Furthermore, after compensation by the coefficient search algorithm, GA, WOA and IWOA, the sensitivities of the compensated measurements decreased by 3 μGal/$\sqrt{\mathrm{Hz}}$, 24 μGal/$\sqrt{\mathrm{Hz}}$, 43 μGal/$\sqrt{\mathrm{Hz}}$ and 47 μGal/$\sqrt{\mathrm{Hz}}$, respectively. Among these, IWOA produced the most significant reduction, corresponding to an improvement of about 50% compared with the uncompensated case. It enhances the sensitivity of gravity measurement while maintaining a small error bar, making it the optimal approach with the best overall compensation performance.

Furthermore, in order to verify the accuracy of the IWOA algorithm in restoring the real vibration of the mirror, a passive vibration isolation system was constructed using Minus-K 50BM-4 (Inglewood, CA, United States). The seismometer was placed above the vibration reduction platform to monitor the vibration of the Raman reflector that was rigidly connected above the seismometer; the structure is shown in Figure 6c. In statistics, the Pearson correlation coefficient measures the linear relationship between two variables. We aim to maximize the Pearson coefficient between the atomic transition probability and the computed phase, which is defined as the normalized covariance of the two variables. When the sweep frequency rate of the atomic interferometer was locked at the center of the cosine ramp (*P* ≈ 0.5), the interference fringes were the most sensitive to the phase change, with the highest sensitivity and a nearly linear relationship between the atomic transition probability and the interference phase (as shown in Equation (3)), enabling a linear mapping between the phase and the gravitational change. To avoid excessive noise in the seismometer caused by the coupling between the horizontal and vertical axes, we simultaneously recorded the seismometer outputs in the V, EW, and NS directions, and expanded the transfer function simplified model from a two-dimensional $\left[K,\tau \right]\;$ (only considering the vertical direction) to a four-dimensional $\left[K_{x},K_{y},K_{z},\tau \right]$ (considering the three-axis directions) parameter model [27], Equation (5) is transformed into the following Equation (20) as

(20) $$\Delta \varphi_{vib}=k_{eff}\cdot \int_{T}^{-T}\left[\frac{K_{x}}{K_{S,x}}\cdot U_{x}\left(t+\tau \right)+\frac{K_{y}}{K_{S,y}}\cdot U_{y}\left(t+\tau \right)+\frac{K_{z}}{K_{S,z}}\cdot U_{z}\left(t+\tau \right)\right]g_{s}\left(t\right)\mathrm{dt}\;$$

where $U_{x}\left(t\right),\;U_{y}\left(t\right),\;U_{z}\left(t\right)$ represent the vertical (z), east-west (x), and north-south (y) vibration voltage signals output by the seismometer. $K_{x},\;K_{y},\;K_{z}$ represent the gain coefficients in the three-axis directions. $K_{S,x},\;K_{S,y},\;K_{S,z}$ represent the nominal sensitivities of the three-axis seismometer. The Pearson coefficient was close to 1, indicating a high correlation between *P* and $\Delta \varphi_{vib}$, thereby demonstrating that the experimental data was highly consistent with the theoretical model and could precisely compensate for the phase noise caused by vibration.

**Figure 13:**
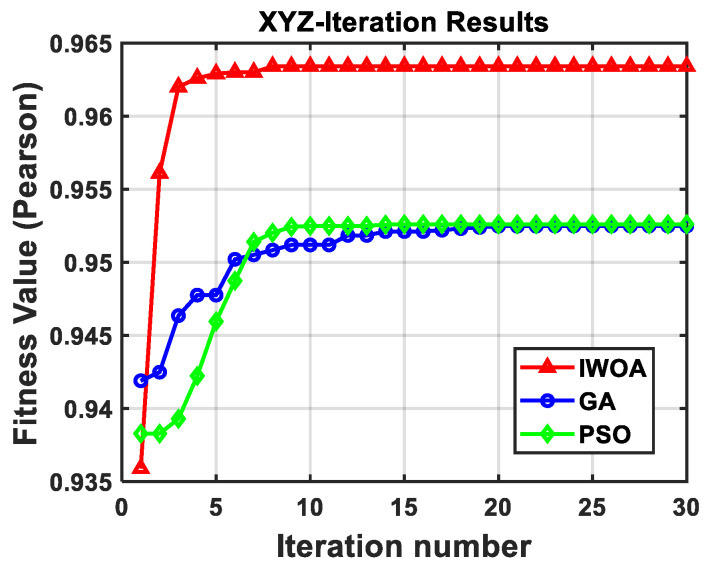
Comparison of different optimization algorithms in optimizing the parameters of the transfer function model. After incorporating the vibration disturbances in the EW and NS directions as weights, IWOA achieved the optimal fitness value after the 8th iterations (best), PSO after the 15th, and GA after the 20th (worst).

The IWOA, PSO and GA algorithms converged at the 8th, 15th, and 20th iterations, respectively. The maximum fitness values were 0.963, 0.952, and 0.952, respectively. From Figure 13, it can be seen that compared with PSO, IWOA can quickly approach the optimal solution while maintaining a high fitness value, achieving a higher local search ability. Compared with GA, IWOA can achieve rapid convergence and has smaller fluctuations after convergence, achieving stronger global optimization ability.

**Figure 14:**
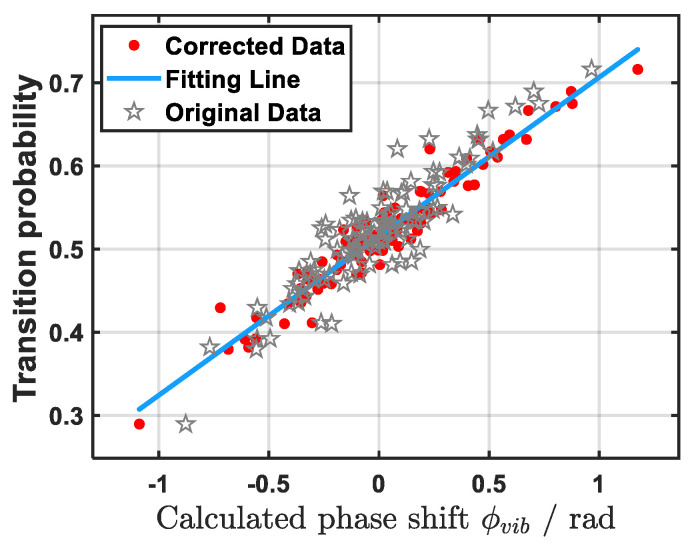
Comparison of results before and after compensation with actual measurement data. Compared to the original data, the corrected data (with IWOA) are closer to the fitting line and show a higher correlation with the atomic transition probability. Fitting line: The data fitting line after compensation, with a correlation coefficient of 0.96.

The comparison diagram before and after vibration compensation is shown in Figure 14. Before compensation, the Pearson coefficient between the atomic transition probability and the calculated vibration phase was 0.914. After compensation, the Pearson coefficient between the two increased by 0.049 to 0.963. As can be seen from the figure, compared with before compensation, the data points after compensation are closer to the fitting line, and the correlation between the calculated phase and the measured transition probability is higher; that is, the compensated phase is closer to the real Raman mirror vibration phase. The experimental record verified the vibration compensation of 20 groups of atomic signals and seismometer signals. The mean of the Pearson coefficient before compensation was 0.918. After using IWOA for compensation, the mean value of Pearson became 0.948, increasing by an average of 0.03, and the maximum could reach 0.98. The overall result was significantly better than that reported by Le et al. (0.94).

**Figure 15:**
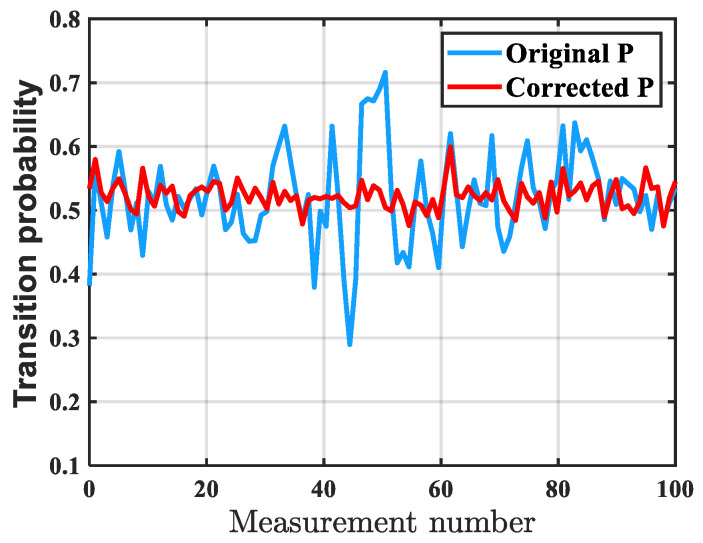
Comparison of atomic transition probability results before and after compensation. The compensated atomic transition probability values are more stable, indicating that after the algorithm correction the interference caused by vibrations has been effectively eliminated.

The comparison of the atomic transition probability results before and after compensation is shown in Figure 15. Before compensation, the peak-to-peak amplitude of the atomic transition probability was 0.43, and after compensation, it was 0.11, reducing by 74% compared with before compensation. Therefore, it is proved that the IWOA vibration compensation algorithm reduces distortion caused by phase misalignment and amplitude mismatch through more accurate delay correction and amplitude calibration, making the vibration phase calculated from the seismometer signal more in phase and in frequency with the fluctuations of the atomic signal and significantly improving the overall stability and consistency.

## 5. Conclusions

To enhance the sensitivity of atomic interferometric gravity measurements, we propose a vibration compensation scheme for the cold-atom absolute gravimeter NIM-AGRb-1, centered on an improved whale optimization algorithm (IWOA). The scheme optimizes the transfer function model to determine the best coefficients, enabling a more accurate estimation of Raman mirror vibrations and thereby achieving precise vibration compensation. The proposed IWOA-based method incorporates a Logistic-LHS hybrid initialization strategy to broaden the global search space and avoid blind zones, employs a sigmoid function and adaptive adjustment mechanism to balance global exploration with local exploitation, and introduces Gaussian-distributed stochastic mutations to prevent premature convergence to local optima. Multiple simulation studies were conducted to validate the method. The results demonstrate that the algorithm accurately identifies the preset gain coefficient K and delay coefficient τ, and the average RMSE of the cosine fitting of the compensated atomic interference fringes was reduced from 165.17 × 10^−3^ to 0.27 × 10^−3^, confirming the effectiveness of the proposed approach. Experimental tests performed in a well-isolated underground laboratory further verified its performance. In a single measurement, the fitted phase parameter uncertainty decreased by 66% after compensation, and the gravity measurement uncertainty reduced threefold without incurring high computational cost. In multiple measurements, the uncertainty of the fitted phase parameter was reduced to below 25 mrad, and gravity measurement sensitivity improved by 50% to 47 $\mu \mathrm{Gal}/\sqrt{\mathrm{Hz}}$. Furthermore, considering the noise influence brought by the horizontal axis, the transfer function model was expanded from $\left[K,\tau \right]$ to $\left[K_{x},K_{y},K_{z},\tau \right]$. After IWOA compensation, the Pearson correlation between the atomic signal and the calculated phase was on average increased by 0.03, with the maximum reaching 0.98.

In summary, the IWOA proposed in this paper demonstrates superior convergence characteristics and optimization capabilities in the vibration compensation problem of atomic interference gravity measurement: On one hand, IWOA can approach the high fitness solution more quickly and maintain smaller fluctuations after convergence, possessing stronger local fine search ability and global optimization stability. On the other hand, this advantage directly improves the restoration accuracy of the real mirror vibration and the phase noise suppression effect, ultimately achieving the improvement of gravity measurement sensitivity with lower computational overhead. The multi-dimensional excellent performance of IWOA indicates that it has greater robustness to changes in the experimental environment. Compared with traditional algorithms (such as PSO, GA, etc.), the IWOA has stronger universality and can better adapt to different experimental conditions and equipment configurations. In the future, we will move further from offline post-processing to real-time vibration compensation and optimize the vibration compensation scheme further for complex vibration environments in outdoor conditions.

## Figures and Tables

**Table 1:** Evaluation of single-shot vibration correction results across various approaches.

	Original	Coefficient	GA	IWOA
Computation time/s	-	76	21	15
Fitted phase parameter uncertainty/mrad	49	27	23	16
Gravity measurement uncertainty/μGal	48	26	22	16

**Table 2:** Evaluation of multiple vibration compensation methods based on experimental data.

	Original	Coefficient	GA	WOA	IWOA
Resolution/(μGal@160s)	6	7	6	3	2
Sensitivity/(μGal/$\sqrt{\mathrm{Hz}}$)	94	91	70	51	47
Error bar/(μGal@160s)	3	3	7	4	3

## Data Availability

The original contributions presented in this study are included in the article. Further inquiries can be directed to the corresponding author(s).
